# “It should just be about sport!”: exploring Italian athletes' perspectives in paralympic media coverage

**DOI:** 10.3389/fspor.2025.1640762

**Published:** 2025-08-22

**Authors:** Athanasios (Sakis) Pappous, Pablo Gómez-Iniesta

**Affiliations:** Department for Life Quality Studies, University of Bologna, Bologna, Italy

**Keywords:** paralympic sport, media representation, disability, framing theory, athlete perception, lived experience

## Abstract

This study explores how Italian Paralympic athletes perceive their representation in the media, shifting the analytical focus from media texts to the lived experiences of the athletes themselves across their sporting careers. Using semi-structured interviews and a hybrid inductive–deductive thematic analysis, the research identifies key themes in how athletes evaluate current media coverage. Findings reveal that while visibility has improved, narratives remain dominated by stereotypical frames—such as the “supercrip” or pity narratives—that marginalise athletic performance. Athletes advocate for more balanced, sport-centered coverage and highlight the role of self-representation through social media in challenging mainstream portrayals. Drawing on theories of framing, representation, and disability models, the study underscores the importance of incorporating athletes’ voices to advance inclusive and accurate media narratives.

## 1 Introduction

Paralympic sport has seen growing media visibility and public interest in recent years, especially following high-profile Games like London 2012 (1–3). With increased coverage of elite disability sport, there is rising awareness of Paralympic athletes in the mainstream media. This growing interest can also be seen in on-demand content platforms, for example, with the documentary Rising Phoenix produced and broadcast by Netflix, which reinforces the normativity of empowerment and narratives such as the superhero or the medical tragedy (4).

However, much of this coverage continues to rely on stereotypical narratives that frame disability in limiting ways. Two dominant frames prevail:

Firstly, the “supercrip” or heroic narrative portrays disabled athletes as superhuman inspirations who “overcome” their impairment through extraordinary feats. For example, Paralympians are often depicted as “heroic individuals who are overcoming their disabilities and providing hope for others” (5). This “superhero” framing can imply, as Shapiro (6) observed, that people with disabilities are only respected when they achieve something deemed extraordinary.

In contrast, media sometimes cast people with disabilities in the role of the pitiable victim, emphasizing tragedy, dependency, or the need for charity. In this framing, a disabled person is presented as an object of pity and in need of assistance, eliciting sympathy and reinforcing the notion that disability is a sad personal misfortune (7). Both extreme representations—the inspirational super-athlete and the object of pity narratives—reflect and perpetuate reductive views of disability, inadvertently reinforcing the notion that disability must be “overcome” or pitied, rather than recognized as a natural aspect of human diversity.

Such stereotypical media portrayals matter because they shape public perceptions of disability and influence how society values people with impairments (8). Scholars have critiqued the supercrip narrative for reinforcing low expectations about disability: praising a person with an impairment for everyday activities or basic independence suggests that simply living one's life is heroic (9, 10). Rather than addressing social barriers or the status of a high-performance athlete, the focus is on disability as an individual obstacle overcome with extraordinary courage.

By perpetuating these narratives and denying them fair representation as professional athletes, the media dehumanizes athletes with disabilities. In this sense, Grue (11) alludes to another media prejudice and quotes Young (12) to talk about the term “inspirational porn” in reference to those representations that exalt everyday achievements of people with disabilities in a condescending way, only to inspire the non-disabled public. A representation of disability in a sentimentalised way for the motivational delight of audiences, which objectifies people with disabilities and reinforces the idea that any achievement of theirs—no matter how everyday—is extraordinary.

### 1.1 Background: contextualizing research on paralympic media representation

Prior research has raised critical questions about the degree to which current media coverage empowers Paralympic athletes, often highlighting the perpetuation of stereotypes and paternalistic views (13). While a substantial body of work has analyzed media portrayals of Paralympic sport—for instance, examining newspaper framing of Paralympic events or the frequency with which athletes are described in terms of disability vs. athleticism—the personal perspectives of the athletes themselves on their career-long media interactions have received considerably less attention.

Previous studies have identified limitations in coverage, including lower news volume compared to Olympic events and framing that prioritizes disability or sensationalism over sporting performance (2, 14–16). However, limited research has explored the perceptions and experiences of Paralympic athletes regarding their empowerment or under-representation in the media.

This gap in research is particularly pronounced in the case of Italy, where academic scholarship on Paralympic sport and media is still in its early stages. Consequently, key questions remain: Do Italian Paralympic athletes perceive the “hero” label as positive recognition, or do they find it patronizing? How do they react to pity or charity framing in stories about them? Addressing this gap is crucial; as Pearson and Misener (17) emphasize, the voices and interpretations of those with lived experience—the athletes—have been largely absent from studies of Paralympic media representation. Without their perspectives, our understanding of disability representation in sport remains incomplete.

This paper addresses this gap by shifting the focus from media representations to athlete perceptions, offering an interdisciplinary contribution at the intersection of sports sociology, communication, and disability studies. Rather than solely analyzing media output, the study explores how Italian Paralympic athletes interpret and respond to media narratives about disability in sport (18). Through qualitative interviews, the research examines how athletes negotiate dominant frames—such as the “superhero” narrative—and how these representations influence their identity, sense of empowerment, and opportunities for more authentic and inclusive self-representation.

## 2 Literature review

### 2.1 Media coverage of paralympic athletes

#### 2.1.1 Disability and sport: identity and representation

Disability theory provides a crucial foundation for understanding how athletes are portrayed in the media. The conceptualization of disability—whether as an individual deficit or a sociocultural construct—significantly shapes the narratives that emerge in media representations. Table 1 outlines the main models of disability, providing a summary of their core characteristics as described in the academic literature.

**Table 1:** Models of disability.

Disability model	Characteristics	References
Medical	Views disability as a defect or illness inherent in an individual. The focus is on diagnosis, cure, and rehabilitation by medical professionals. Disability is seen as a personal tragedy or problem to be fixed, with “normalization” as the goal.	(19, 20)
Social	Disability results from external barriers—social, environmental, or attitudinal—rather than from the individual's impairment. The model calls for systemic changes to remove obstacles and promote accessibility, inclusion, and equal rights. It shifts responsibility from the individual to society.	(21, 22)
Sociorelational	Disability is produced through the dynamic interaction between the individual and their environment. It integrates social oppression and the lived, embodied experience of impairment. It acknowledges psycho-emotional impacts (e.g., stigma, marginalization) alongside structural barriers, emphasizing relationships and context.	(23–25)
Biopsychosocial	Disability emerges from the complex interplay of biological, psychological, and social factors. As outlined in the WHO's ICF framework, it recognizes impairment but also considers mental health, personal coping strategies, and societal context. It promotes a holistic, multi-dimensional view of disability and functioning.	(26)

Source: Own elaboration based on academic literature.

Sport occupies a unique and sometimes paradoxical place in the politics of disability and identity:

On one hand, athletic participation can be a powerful source of empowerment and positive identity for people with disabilities: Paralympic athletes often describe sport as a realm in which they can define themselves beyond societal prejudices, proving their skills and challenging assumptions about what disabled people can do (27). Research has shown that successful Paralympians can become important role models, instilling a sense of possibility and pride in others with disabilities. Berger's (28) study found that accomplished athletes served as exemplars for younger disabled individuals, providing “important role models for younger athletes to emulate”. In this way, sport can help counteract stigma by showcasing athletes with disabilities as competent, skilled, and resilient. The visibility of Paralympians excelling in competition may encourage a broader rethinking of disability as compatible with strength and achievement, rather than weakness.

On the other hand, the representation of disability in sport is fraught with potential pitfalls. Media coverage of Paralympic sport often emphasizes human-interest angles—personal backstories of injury or illness, or narratives of struggle—sometimes at the expense of recognizing athletic performance and competitive context. Athletes with disabilities frequently find that their public identity is mediated through lenses of inspiration or pity, rather than simply being seen as athletes. The International Paralympic Committee itself has advocated portraying Paralympians as “athletes first and individuals with an impairment second” to normalize their status (29). This reflects a desire to align representation with a social model ethos, focusing on the person's role as an athlete. Yet, studies indicate that audiences and media still often focus on the disability aspect. As Hellwege and Hallmann (30) observed, despite the IPC's official stance, the general public's primary focus tends to remain on athletes' impairments rather than purely on their athletic accomplishments.

This tension means that Paralympic athletes navigate complex identities in media narratives. Being celebrated as “inspirational” can be double-edged: it brings praise and attention but can also feel patronizing or othering. Athletes have reported mixed feelings about the supercrip label—it can be empowering for some, providing visibility and challenging outright negative stereotypes (31), yet it can also set them apart unrealistically or pressure them to embody a heroic ideal. Conversely, being framed as objects of pity is often rejected by athletes themselves, as it undermines their hard-earned sporting achievements and autonomy. In disability sport scholarship, there is a recognition that “fighting negative stereotyping of disability with another stereotype” (like the supercrip hero) is problematic in the long run (32). True empowerment in representation would mean showing Paralympians as multifaceted individuals—as competitive athletes, as people with everyday lives, and as neither superheroes nor objects of charity.

Some media and journalists have started to present Paralympic athletes simply as high-performance athletes, focusing on the sport itself (marks, tactics, competition) and mentioning disability only as a contextual fact, without special emotional connotations (33). This shift is in line with the demands of the independent living movement and athletes themselves, who advocate for a more normalised and respectful representation. Furthermore, the rise of social media has allowed many Paralympic athletes to self-represent themselves and share their stories directly, without going through the filter of traditional media, which diversifies the narratives available to the public (34).

Despite these advances, research suggests that quantitative and qualitative differences in media coverage persist between Paralympic sport and mainstream sport. In terms of quantity, attention to the Paralympics tends to be concentrated around the Games period and declines significantly in the intervening years (2). In terms of quality, it has been observed that certain disabilities or sport disciplines receive preferential treatment in terms of heroism, while others may be ignored or treated with morbidity (15).

These findings underline the importance of listening to athletes: to understand how they value the coverage they receive and what aspects they would like to see transformed.

#### 2.1.2 Framing theory in media coverage

To analyze media representation of Paralympic athletes, we draw on framing theory from communication studies. Framing theory examines how the media selectively emphasizes certain aspects of reality to shape audience understanding. A frame (35) is essentially the central organizing idea or storyline that provides meaning to an unfolding strip of events (in this case, stories about athletes with disabilities). As Entman (8) famously defined: “Framing essentially involves selection and salience. To frame is to select some aspects of a perceived reality and make them more salient in a communicating text” (p. 52).

By highlighting particular facts, values, or themes, media frames promote specific interpretations and evaluations of an issue. For example, a news article can frame a Paralympic gold medalist's story as one of personal triumph over adversity—selecting details about medical rehabilitation and personal grit—or alternatively frame it as a story about inequities and the need for better support—focusing on societal barriers the athlete navigated. The selection of which narrative to tell and the salience given to disability vs. athletic achievement will cue audiences to understand the athlete in a certain way (hero, victim, regular competitor, etc.).

Frames operate through subtle but effective mechanisms: the language used, the characters quoted, the context provided and even the visual resources chosen are part of the frame (8). For example, when covering a Paralympic competition, a media outlet may opt for a frame focused on sporting achievement (talking about marks, records, competitive strategy) or, alternatively, a frame focused on the athlete's biography (emphasising their injury or illness and their supposed “overcoming”). Each approach leads the audience towards a different interpretation: the former normalises the athlete as an elite athlete, while the latter places the athlete in a narrative of exceptionality due to disability. Framing theory reminds us that no journalistic narrative is completely neutral; they all highlight certain values and perspectives (36).

In research on media and disability, several typical framings have been identified. In addition to the supercrip and pity frame mentioned above, Haller (37) documents others such as the medical frame (which focuses on diagnoses, treatments or clinical terminology), the rights frame (which highlights issues of equality, accessibility and claims of people with disabilities) or the paternalistic frame (which presents people with disabilities as dependent or infantilised). Each of these frames carries ideological implications: for example, the rights frame is often associated with a discourse of social justice and political change, while the paternalistic frame reinforces the notion of persons with disabilities as objects of care rather than autonomous agents (13).

For the specific case of Paralympic sport, previous studies employing framing analysis have revealed interesting tensions. Solves et al. (2), in their analysis of Spanish media during Beijing 2008, found that news stories varied between a patriotic-sports framing (celebrating medals and national achievements) and humanitarian or emotional framing (highlighting life stories). Similarly, Buysse and Borcherding (38) observed in the US press a gradual evolution from tragedy-focused framing to more sporting framing, although still mixed with inspirational language. These results suggest that the media framing of Paralympic sport is in transition, influenced both by societal changes (increased disability awareness) and the professionalisation of Paralympic sport itself. Nonetheless, narratives of inspiration and personal heroism remain a prevalent frame when discussing athletes with disabilities, which warrants further critical examination of what effects this has on public perception and on athletes themselves.

### 2.2 Representation and meaning-making

Media framing connects closely with questions of representation—how meaning is produced and understood through media. Stuart Hall's theory of representation provides a valuable lens for this study, particularly his encoding/decoding model (39). Hall posited that media messages are encoded with meanings by producers (journalists, editors, etc.), but those messages are not received in a uniform way by audiences. Instead, audiences decode messages according to their own frameworks of understanding, which can lead to interpretations that align with, negotiate, or oppose the intended meaning. In Hall's view, meaning is not fixed or guaranteed by the sender; the audience is not a passive recipient but an active interpreter. He argued that “the message sent is seldom (if ever) the one received”, because people interpret media through their own social contexts and identities.

Hall (40) outlined three hypothetical positions that an audience member might take in decoding a media text: a dominant (or preferred) reading where the viewer accepts the intended meaning, a negotiated reading where the viewer partly accepts the frame but modifies it based on their own perspective, and an oppositional reading where the viewer recognizes the intended frame but actively resists or rejects it, substituting an alternative interpretation.

In the case of Paralympic media narratives, a dominant reading by a general audience might be to admire a portrayed athlete as inspiring (accepting the supercrip framing as positive). A negotiated reading might be to appreciate the athlete's skill but also feel the media exaggerated the “overcoming” aspect. An oppositional reading could be to critique the story for focusing on disability at the expense of athletic achievement, thus rejecting the media's framing.

Paralympic athletes themselves constitute a unique and crucial audience for media representations about them. They occupy an insider-outsider position: as subjects of representation, they have intimate knowledge of the lived experience behind the media stories, but as consumers of media, they also must interpret narratives that others construct about their lives and performances. Hall's concepts suggest that athletes might decode media portrayals in ways that differ from the general public's reception. For example, an athlete might resist the sentimental pity narrative, finding it stigmatizing, or they might challenge a heroic narrative as glossing over the real barriers they face.

Alternatively, some athletes might embrace certain media frames—for instance, feeling proud that their story is used to inspire others—but still negotiated he terms of that portrayal (perhaps by reframing inspiration in terms of hard work and sportsmanship rather than “overcoming disability”). Indeed, previous qualitative research suggests that many athletes with disabilities have nuanced views on how they should be portrayed: they appreciate the press interest but would like to see more emphasis on their concrete sporting achievements and less on melodramatic narratives (29).

Using Hall's theory of encoding/decoding in this study allows to conceptualize the interviewees (the athletes) as active interpreters of representation. The media may encode Paralympic coverage with dominant frames (such as triumph-over-adversity), but athletes are not simply absorbing these messages—they are responding, contesting, and reinterpreting them through their own lens as competitors and as people with disabilities. This theoretical perspective underlines the importance of listening to the athletes' own interpretations. It reminds that representation is a two-way process: the power of media framing is significant, but so is the agency of audiences to make meaning.

While Hall's model highlights the interpretative agency of the audience in decoding media texts, it does not fully account for cases in which individuals are both subjects of representation and active producers of media. In the context of Paralympic sport, athletes engage not only in decoding dominant portrayals but also in actively responding through self-representation. This includes the use of personal platforms—such as interviews, social media, or advocacy—to challenge dominant frames and construct alternative narratives that reflect their preferred identities (e.g., “athlete first”). These practices resonate with Foucault's notion of technologies of the self: the ways in which individuals, within structures of power, work to shape their identities through self-directed discursive strategies (41). Integrating self-representation into Hall's framework thus provides a more comprehensive understanding of how power and identity are negotiated through both the reception and production of meaning.

To synthesise the main media frames that shape representations of Paralympic athletes, Table 2 offers a comparative overview of common framing strategies identified in previous research. These frames reveal distinct ways in which disability and athletic identity are constructed through media narratives.

**Table 2:** Media framing of paralympic athletes: narrative features and representational impact.

Media frame	Key features	Effects on representation
Supercrip/Inspiration	Focuses on overcoming disability through extraordinary achievement.	Portrays athletes as heroic but risks reinforcing unrealistic expectations or otherness.
Pity/Charity	Emphasises tragedy, dependence, and emotional appeal.	Frames athletes as victims, reinforcing vulnerability and passivity.
Medical	Highlights impairment, diagnosis, or recovery.	Reduces athletes to their condition, ignoring sporting identity.
Rights-Based	Centres on equality, inclusion, and access.	Positions athletes as political subjects fighting for justice and representation.
Sport-Performance	Focuses on results, tactics, training, and competition.	Frames athletes primarily as elite competitors, minimising reference to disability.
Paternalistic	Uses infantilising or protective tones.	Depicts athletes as needing care rather than agency or professionalism.
Self-Representation	Content created or controlled by the athletes themselves.	Enables more authentic, multidimensional representations beyond traditional media filters.

Source: Own elaboration based on academic literature.

In sum, this study draws on this interdisciplinary theoretical framework: disability models provide the lens for understanding the ideological implications of different narratives and identities; framing theory provides tools for analysing the media construction of these narratives; and Hall's perspective on representation and decoding helps to place the voice of athletes at the centre of the analysis.

## 3 Methodology

### 3.1 Research design

An exploratory qualitative approach was chosen, suitable for an in-depth investigation of the subjective perceptions and experiences of a specific group (42). A cross-sectional study design was carried out, based on semi-structured interviews with Italian Paralympic athletes. This method allows for capturing participants' personal narratives and exploring emerging themes with flexibility, while ensuring that certain key topics related to the research question are addressed by all. The semi-structured interview also provides an effective comparative model for the analysis of multiple interviews, allowing for individual variation while maintaining thematic relevance across participants (43).

The overarching question guiding the study was:

How do Italian Paralympic athletes perceive current media representations of Paralympic sport, and what recommendations do they suggest for fostering more inclusive and accurate portrayals?

The interviews were designed to explore key dimensions of media representation, such as framing strategies, the focus of coverage (disability vs. athleticism), and athletes' agency in shaping their own narratives. This exploratory study aims to investigate how athletes view current media portrayals, specifically concerning how they evaluate the balance between coverage of personal stories/disability and sporting achievement, and to explore their suggestions for future media coverage of Paralympic sport. Furthermore, the study explores how athletes with greater media exposure engage with dominant framing strategies, including “supercrip” and “pity” narratives. Finally, it examines how athletes active in self-representation—through social media or public advocacy—challenge stereotypical portrayals and promote alternative narratives that foreground personal agency and competitive identity.

#### 3.1.1 Italy: a relevant case for analyzing paralympic media dynamics

Italy provides a relevant case for understanding the intersection of media and the Paralympic movement, notably as the host of the first official Paralympic Games in Rome in 1960 and a nation with increasingly successful delegations. This historical significance, combined with consistent athletic success and evolving media landscapes, makes Italy a compelling context for this study. The evolution of Italy's Paralympic success is evident in recent editions (44): at the London 2012 Games, 98 Italian athletes won 28 medals (including 9 gold), while in Paris 2024 the Italian team grew to 135 athletes and achieved 71 medals (35 gold). This athletic progress reflects growing institutional investment and the increased visibility of Paralympic sport within Italian society.

This athletic success, alongside growing institutional investment, has coincided with a marked growth in Paralympic coverage in Italy. Rai 2 positioned itself as the “Paralympic channel,” achieving an average daily audience of 611,000 viewers, with peaks of 1.2 million—surpassing the 400,000 average registered during the Tokyo 2020 Paralympic Games (45). This increase in viewership signals a broader cultural shift, indicating that Paralympic sport is gaining traction beyond niche audiences. Rai's coverage was further amplified by Rai Sport and a strong digital presence, contributing to the normalization and celebration of Paralympic achievements in mainstream discourse.

Younger demographics have played a particularly important role in this transformation. According to Rai data, 25% of the television audience for the Paralympics was under the age of 35, suggesting that new generations are increasingly engaging with inclusive sport narratives. Furthermore, social media has significantly contributed to this trend: the 2024 Games generated 5.5 million interactions across digital platforms, with Instagram as the most-used channel (45). The engagement of younger audiences and the prevalence of social media highlight the evolving nature of Paralympic media consumption in Italy.

In conclusion, Italy offers a powerful case study for examining how athletic excellence, institutional support, and evolving media landscapes converge to shape cultural understandings of disability, inclusion, and representation.

#### 3.1.2 Participants

Participants comprises 17 elite Italian Paralympic athletes, selected for their high-level achievement in a range sports (e.g., athletics, swimming, team sports). The sample includes both male and female athletes with extensive experience and with many having achieved multiple Paralympic medals from 1988 up to Paris 2024 (see Table 3). All participants gave informed consent to partake in the study, and ethical approval was obtained through the relevant university ethics committee prior to data collection.

**Table 3:** Participants overview.

ID	Gender	Age	Discipline	Paralympic games participation	Medals in paralympic games
A.1	Female	35	Athletics	2012, 2016, 2020, 2024	3 gold medals, 3 silver medals
A.2	Female	43	Athletics	2016, 2020, 2024	3 bronze medals
A.3	Female	32	Canoeing	2024	No medals
A.4	Male	25	Cycling	2024	No medals
A.5	Male	35	Weightlifting	2024	No medals
A.6	Male	25	Swimming	2020, 2024	1 gold medal, 2 bronze medals
A.7	Female	39	Triathlon	2020, 2024	1 silver medal
A.8	Female	21	Equestrian	2020, 2024	No medals
A.9	Male	27	Judo	2024	No medals
A.10	Male	48	Cycling	2000, 2008, 2012, 2016, 2020, 2024	2 silver medals, 1 bronze medal
A.11	Male	37	Ice Hockey	2010, 2014, 2018, 2022	No medals
A.12	Female	50	Sitting Volley	2020, 2024	No medals
A.13	Female	53	Cycling	2020, 2024	1 bronze medal
A.14	Female	30	Rowing	2020, 2024	No medals
A.15	Female	54	Athletics Cycling Nordic Skiing	1988, 1992, 1996, 2000, 2004, 2006, 2008, 2010, 2014, 2016, 2020, 2024	3 gold medals, 4 silver medals, 7 bronze medals
A.16	Male	30	Swimming	2012, 2016, 2020, 2024	4 gold medals, 1 silver medal
A.17	Female	51	Athletics	2024	No medals

The anonymity of the interviewees was guaranteed, and for the purposes of analysis, each participant was identified using an alphanumeric code ranging from A.1 to A.17. This measure was taken to protect the privacy of the athletes and to ensure that their responses could be expressed freely, without concern for public or institutional exposure.

### 3.2 Instruments and data collection procedure

A semi-structured interview script was designed specifically for the aims of the study. This script covered the following main topics or sections:

• **Media consumption and information habits:** where and how athletes usually get informed about sports news (TV, newspapers, social media), and whether they feel that traditional media pay enough attention to Paralympic sport.
• **Personal experience of coverage:** Athletes recounted examples of how the media have covered their sport performances throughout their careers, focusing on general patterns rather than single events. Perceptions of quantity of coverage (visibility) and quality of coverage were addressed here.
• **Perceived frames and narratives:** We explored whether athletes identify patterns in the way they are portrayed. For example, whether they notice emphasis on their disability as opposed to their sporting achievements, use of sensationalist or emotive language, etc. They were asked how they feel about “success story” or other types of framing.
• **Stereotypes and misconceptions:** Going deeper into the above, possible stereotypes (positive or negative) in the coverage were discussed. Also, whether they consider that there are important aspects of their stories that are often ignored by the media.
• **Interactions with journalists and control of the narrative:** They were asked about their interactions with media professionals (have they had the opportunity to express how they wanted to be presented, have they corrected any information, and have they tried to actively influence their stories?) Also, whether they have actively tried to influence their own representation, for example through social media.
• **Recommendations for improved representation:** Towards the end, athletes were invited to provide concrete suggestions for the media for a fairer, more accurate and respectful future coverage of Paralympic sport and its protagonists.

The interviews were conducted between June and December 2024. They were initially planned to be face-to-face, but given the geographical availability of participants, the majority were conducted by secure videoconference (Microsoft Teams). Each interview lasted approximately 30–45 min. All were conducted by the main researcher (fluent in Italian and experienced in qualitative interviews), supported by an assistant. With the permission of the participants, the interviews were audio-recorded for later verbatim transcription.

During the interview, the tone was conversational, allowing the athletes to elaborate on the topics they considered relevant, while the interviewer made sure to cover all the points in the script. After each interview, a verbatim transcript was made in Italian. The transcripts were checked against the audio recordings to ensure accuracy.

Rather than focusing on reactions to a specific media event, the interviews were designed to explore athletes' reflections on media coverage throughout their sporting careers. This approach allowed us to capture their lived experiences and personal interpretations of recurring narratives, stereotypes, and representational dynamics in both traditional and digital media over time.

### 3.3 Thematic analysis approach

The study utilizes a hybrid inductive–deductive thematic analysis, combining data-driven coding (allowing themes to emerge inductively from the data) with theory-driven coding (using a theoretical framework and literature to deductively examine specific concepts) to ensure a thorough and balanced analysis of qualitative data (46, 47). This approach, supported by guidelines from Fereday and Muir-Cochrane (48) and Braun et al. (49), enhances transparency and rigor by systematically integrating qualitative data with theoretical constructs, yielding a rich, credible thematic structure grounded in participants' lived experiences and informed by existing theory (48, 49).

While this study primarily employs a qualitative thematic approach, it also integrates a quantitative element by measuring code frequencies across interviews. This approach facilitates the identification of dominant themes and visualization of the relative salience of specific narratives. Although frequencies do not provide statistical generalizability, they enhance transparency and allow for comparisons in theme development (50, 51). Integrating this quantitative perspective strengthens the analysis by grounding interpretive insights in empirical data.

This hybrid approach is particularly suited to the study, enabling both the inductive capture of athletes' descriptions of their experiences and media interactions, and the deductive examination of those descriptions through representation theories and disability models. This iterative process enhances rigor and validity by capturing both the richness of the data and the influence of prior concepts, ensuring the analysis remains connected to the study's theoretical foundation and research questions.

#### 3.3.1 Coding process

The coding process employed a hybrid inductive-deductive approach. Initially, an iterative, line-by-line reading of interview transcripts enabled open, inductive coding, identifying recurring ideas, concepts, and emotions, including nuanced perspectives and anecdotes that did not always align with existing theoretical categories (52).

In parallel, a deductive coding framework was developed, informed by the study's theoretical foundations (media framing, representation theory, and self-representation). *a priori* codes, derived from relevant literature and the research questions, included categories such as “Stereotyped Media Narratives” (with subcodes like supercrip/inspiration, pity/charity, and tragedy-focused storytelling), “Visibility in Media” (e.g., sporadic attention and comparison with Olympic athletes), and “Suggestions for Improving Coverage” (e.g., focus on performance and avoiding stereotypes). Aspects of Hall's encoding/decoding positions and self-representation theory (e.g., resistance to media portrayal) were also included.

The analytical process remained flexible, allowing inductive insights to refine or expand the deductive framework, contributing to a more nuanced and participant-centred interpretation of dominant media frames. ATLAS.ti facilitated the systematic organization, retrieval, and comparison of coded segments, supporting collaborative development of the coding schema.

Subsequently, major themes were constructed through iterative comparison and interpretative analysis, capturing key elements in athletes' accounts. Analytical rigour and coherence were ensured through joint review and validation of all codes and subcodes by the two researchers involved in the study. The data gathered was subsequently examined considering the theoretical framework introduced earlier and is discussed in the following sections.

## 4 Results

The thematic analysis yielded a rich set of themes capturing Italian Paralympic athletes' perspectives on media representation. Through a hybrid inductive-deductive approach, identified 4 major themes were identified, each with several subthemes for a total of 16 subcodes. These encompassed topics anticipated from prior research (e.g., Role of social media, Suggestions for Improvement) as well as emergent concerns (e.g., specific Stereotyped Narratives and a visibility of paralympic sport in media).

Table 4 presents all main themes and subthemes along with their absolute frequencies (number of coded references across all 17 interviews), illustrating the salience of each topic in the athletes' narratives. In the sections that follow, each theme is described in turn, supported by example quotes from athletes A.1 through A.17.

**Table 4:** Major themes, subthemes, and absolute frequency of coded references.

Code	Subcode	Frequency
Visibility of Paralympic sport in media	Satisfaction/improvement in recent years	8
	Sporadic media attention	12
	Comparative treatment vs. Olympic athletes	17
	Desire for authentic representation	12
Stereotyped media narratives	“Pity”/charity frame	12
	“Superhero” inspirational narrative	12
	Focus on tragedy/disability story	10
	Questioning athletic legitimacy (“not real sport”)	6
Role of social media (self-representation)	Enhancing public visibility and reach	1
	Tools for identity control and narrative framing	10
	Limitations of social media	5
Suggestions for media improvement	Learn about the sport: “do your homework”	4
	Focus on Athletic performance	16
	Avoid stereotypical narratives	14
	Ensure equal and consistent coverage.	5
	Engage with athletes and tell holistic stories.	5

### 4.1 Visibility of paralympic sport in media

#### 4.1.1 Satisfaction/improvement in recent years

Several athletes acknowledged that media visibility has improved over time throughout their careers. Notably, multiple interviewees mentioned a positive trend in the last decade. “*There has been a significant increase in coverage, I think in 2020”* (A.5) said one, crediting the most recent Games with unprecedented exposure on major TV channels. Similarly, one long-time Paralympian noted he had “seen an evolution over the years” and a “*total change in the media… around the Paralympics after London* [2012]*”* (A.10), emphasizing how far visibility has come.

#### 4.1.2 Sporadic media attention

Nearly all athletes discussed the extent of media coverage their sport receives, often contrasting the surge of attention during the Paralympic Games with the quiet periods in between. Many noted that broad media attention “spikes every four years” and then largely disappears. As one athlete put it, “*Unfortunately it only happens every four years”* (A.11), referring to the intense coverage during the Paralympics as the only time Paralympic athletes truly break into mainstream media.

Participants often referenced the breakthrough of broadcasts on national prime-time television as a sign of progress. Nonetheless, outside the Games themselves, coverage was described as inconsistent or lacking. Athletes shared that between Paralympic cycles, their competitions and achievements receive minimal attention—especially compared to Olympic sports. In sum, the quantity of coverage was seen as improving during Paralympic events (some called recent Paralympic media coverage “very positive”), but athletes remain concerned about the sustainability of visibility in non-Games years.

#### 4.1.3 Comparative treatment vs. olympic athletes

Another strong theme was the perceived inequity in coverage and recognition compared to Olympic (able-bodied) athletes. All 17 Paralympians were keenly aware of differences in media treatment. They pointed out that Olympic athletes' successes typically receive far more fanfare and consistent media presence, whereas Paralympic athletes often feel like a side story except during the Paralympics. One athlete lamented that “the spotlights only turn on [us] every four years, alternating” with the Olympics (A.15), underlining that continuous attention is reserved for their Olympic counterparts. Several athletes argued that their accomplishments should be valued on par with Olympians'. “[It is] not any less important than [for] able-bodied people,” one interviewee emphasized (A.9), rejecting the notion that Paralympic sport is of lower significance.

Others noted that even when media do cover Paralympic successes, they tend to treat them as human-interest pieces rather than front-page sports news. For example, one gold medalist noted that while he did make the “*prima pagina”* (front page) after a major Paralympic win, such attention was fleeting and “exceptional,” whereas Olympic champions routinely command headlines (A.6). Athletes A.14 and A.4 also discussed how financial support and sponsors are much harder to attract due to this limited media exposure—creating a vicious cycle in which a lower media profile translates to fewer resources for Paralympic sports. Across the interviews, there was a clear call for equal respect and coverage: athletes want the media (and public) to see Paralympians as athletes of the same stature as Olympians, not as novelties or secondary figures.

#### 4.1.4 Desire for authentic representation

Several athletes expressed a clear desire for more authentic, sport-centred media representation. Rather than ignoring their disability, they urged journalists to contextualise it without making it the central narrative. As A.3 stated, they want to be “seen more as athletes and less as people with disabilities.” Participants emphasised that they are professional athletes who happen to have a disability—not individuals doing sport as therapy. They suggested that media should focus on performance metrics, tactics, and training like with Olympic athletes, instead of highlighting prosthetics or “overcoming” stories. A.3 proudly described Paralympians as “*atleti di altissimo livello”* (athletes of the highest calibre), calling for coverage that reflects that status. Authentic framing, they argued, would promote respect, normalise their image as elite athletes, and help dismantle stereotypes over time.

### 4.2 Stereotyped media narratives

**Figure 1:**
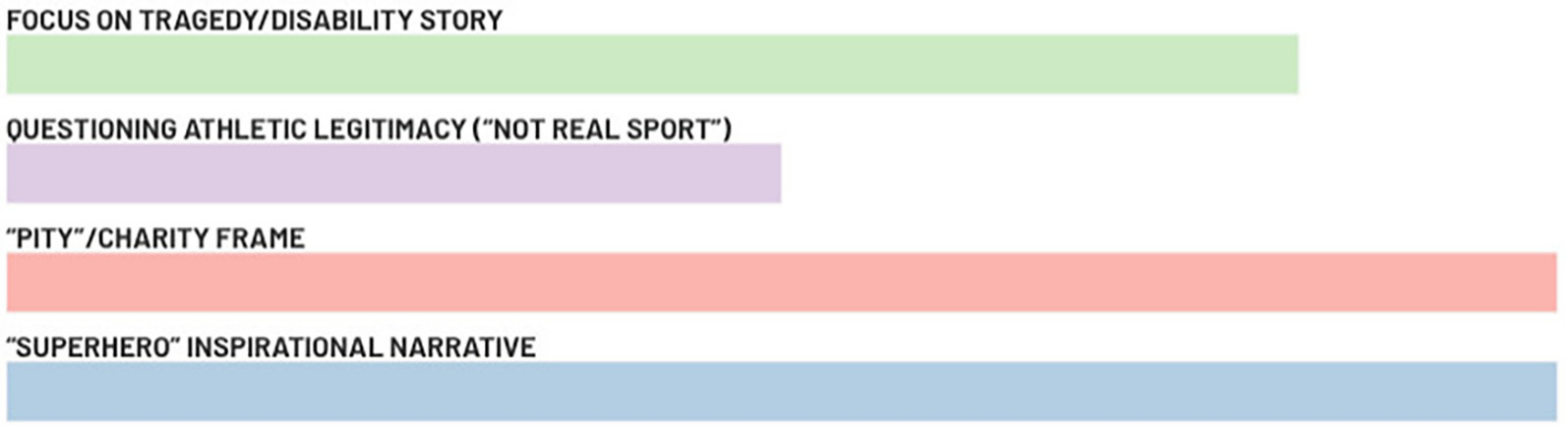
Frequency of stereotyped narratives identified in athletes' responses.

Athletes were highly attentive to how they are portrayed in the media, not just how often. They identified several recurring media frames or narrative patterns that they find problematic based on their cumulative experiences over time. As shown in Figure 1, these include portrayals that evoke pity, those that cast Paralympians as inspirational “superheroes,” a focus on personal tragedy/disability stories, and sometimes even questioning the athletic legitimacy of Paralympic sport. All these stereotyped narratives—whether casting athletes as pitiable victims or as superhuman inspirations—were seen as forms of misrepresentation that the Paralympians hope to see reduced. They much prefer to be covered as multi-faceted athletes, neither saints nor objects of sorrow. As one interviewee summarized, the goal is to be “seen more as athletes and less as people with disabilities” (A.3). This desire for more authentic representation underpinned many of their comments on media portrayal.

#### 4.2.1 Pity/charity frame

Many athletes expressed frustration with coverage that emphasizes disability to elicit sympathy. They observed that some journalists focus on the athlete's impairments or hardships in a patronizing way. One athlete noted that the media often find “the pity aspect is more intriguing—like ‘poor thing, he's missing a leg’” (A.2). Such depictions frame the individual as an object of charity or sorrow rather than a competitive athlete. The interviewees resented being the subject of “pietismo” (pity)—stories that highlight how unfortunate their disability is, instead of their skills or results. This pity framing was described as reductive and outdated, yet, as one Paralympian observed, it still seems to “fascinate” certain audiences and reporters.

#### 4.2.2 “Superhero” narrative

The opposite extreme—portraying Paralympians as superhuman heroes—was equally prevalent and critiqued. Several athletes mentioned being frequently described as “*eroi*” or “*supereroi*” (heroes/superheroes) in media stories over the course of their careers. While one athlete acknowledged she didn't mind the label and “took it with all [her] heart” (A.2), the majority pushed back on this narrative. “We’re not superheroes; we’re just people,” one Paralympian flatly stated (A.7), reflecting a common sentiment that the media often unrealistically glorify athletes with disabilities. Another athlete pointed out that “it's easy to lump us into the superhero category, but then one should look at the rights we have, [and] the difficulties a child without a leg faces…” (A.1). This remark highlights that feel-good “supercrip” labels can gloss over real-world challenges—celebrating athletes as inspirational figures while ignoring the inequities and barriers (e.g., access to sport) that disabled people continue to face. In short, superhuman narratives, even if intended to be positive, made many athletes uncomfortable because they emphasize disability (the idea that one “overcame” it) rather than treating Paralympians' achievements as the result of talent and hard work like any other athlete's.

#### 4.2.3 Focus on tragedy/disability story

Athletes also noted that media coverage is often overly fixated on their personal backstory of disability, sometimes at the expense of discussing the sporting side. Many interviewees recounted that journalists' questions tend to center on how they became disabled or the challenges of living with disability. One athlete gave an example: reporters inevitably ask why she is disabled or “what happened”—but “it should end there” (A.15), implying that the media should not dwell on the impairment once basic context is given. Others echoed that sentiment, saying they are tired of every interview starting with the story of their accident or illness. While they understand there is public curiosity, the athletes prefer that after briefly addressing “what happened,” the conversation move on to their current training, performance, and goals. They pointed out that Olympic athletes are rarely, if ever, asked about deeply personal or traumatic history in sports interviews—yet Paralympians often face more intrusive or pathos-driven questions. A few athletes noted that journalists sometimes appear unsure how to talk to a disabled athlete, oscillating between being overly delicate or overly sensational. One interviewee (A.13) reflected that in the past “diversity has always scared [journalists] a bit… causing difficulty in terms of the words to use or the direct approach with the Paralympic athlete.” However, she and others observed that this is slowly changing as media become more accustomed to covering Paralympians. The clear consensus was a desire for coverage that does not center on the disability origin story or dramatize the athlete's life, but rather treats their athletic journey with the same normalcy as any other athlete's career.

#### 4.2.4 Questioning athletic legitimacy (“not real sport”)

A smaller but significant concern was the implicit (or sometimes explicit) questioning of Paralympic sport's competitiveness. Some athletes felt that portions of the public—and by extension some media narratives—do not regard Paralympic events as “real” sport on par with able-bodied competitions. Reflecting on their experiences over time, one athlete illustrated this attitude by quoting what a layperson might say: “Oh, poor things, they've gone to do their little race?” (A.16). This remark captures a patronizing view that Paralympic competitions are just a “*garetta”* (a “little race”) or a token participation event, rather than high-performance sport. Such perceptions deeply frustrate the athletes. They argued that the media should counter, not subtly reinforce, the notion that Paralympic athletes are not serious athletes. As one swimmer pointed out, “we are athletes 360°” (A.16)—dedicating their lives to training and excelling in sport. Several interviewees noted that when coverage focuses too much on the disability or inspirational angles, it can unintentionally feed the idea that spectators should watch Paralympians out of compassion or curiosity, rather than out of interest in sport competition. The athletes want the media to treat Paralympic results with the same athletic seriousness as any sports achievement, to dispel the lingering stereotype that it's not “real” competition.

### 4.3 Role of social media and self-representation

A notable theme was the growing impact of social media on athletes' public image and their ability to represent themselves. Many Paralympians have turned to platforms like Instagram and Facebook to share their journey, celebrate successes, and connect with fans—sometimes filling the void left by limited mainstream coverage. Several athletes described social media as a double-edged sword. On one hand, it offers a powerful avenue for self-promotion and narrative control. “I got so many messages on Facebook and Instagram… the followers increased,” said one medalist, recalling the wave of support online after her Paralympic performance (A.12). Through personal posts, athletes can highlight aspects of their lives that traditional media might overlook—for example, their daily training routine or behind-the-scenes team camaraderie—thereby painting a fuller picture of themselves as athletes. Some have been able to build substantial followings and even attract sponsorships via social channels. Indeed, a few interviewees noted that social media greatly amplifies their visibility: news about their wins or records can spread quickly online even if TV news barely mentions it.

On the other hand, athletes also acknowledged challenges with social media. Not everyone is equally enthusiastic about maintaining an active online presence. “I'm not very into social media compared to others,” admitted one athlete (A.8), indicating that some prefer to keep a lower profile or find constant posting tedious. There can be pressure to engage frequently and to present a positive image consistently—an extra task on top of training. A couple of participants mentioned encountering negative or ignorant comments on social platforms. One athlete pointed out that social media enables people to voice criticisms or stereotypes more freely—“through social [media] we witness… those who don't want to say things to your face will do so indirectly” (A.17)—highlighting the risk of exposure to trolls or insensitive remarks. Despite these issues, the general view was that the benefits outweigh the downsides. Social media allow Paralympians to bypass traditional gatekeepers and directly educate the public. As a result, many athletes take an active role in managing their social media accounts, seeing it as an extension of their athletic brand. Some do so on their own, while a few noted they have assistance or a strategy for handling fan engagement. In summary, social media has become an important tool for Paralympic athletes to shape their own representation, celebrate their identity as athletes, and rally support—even as they navigate the new demands that come with that visibility.

### 4.4 Suggestions for improving media representation

**Figure 2:**
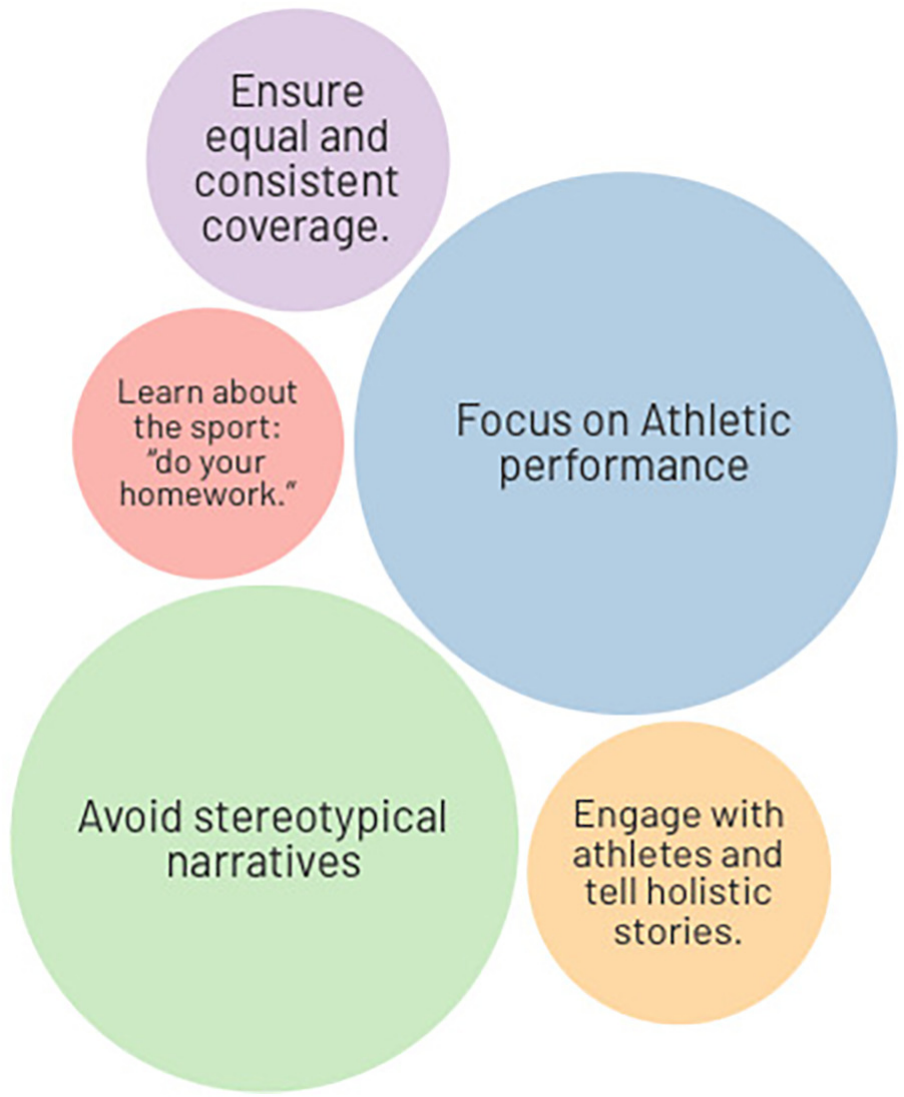
Frequency of athletes' recommendations for improving Paralympic media coverage.

In the final part of each interview, athletes were invited to give advice by imagining a “media guide” for journalists covering Paralympic sport. Their recommendations were strikingly consistent. In essence, the athletes want journalists and media outlets to improve both the quantity and quality of Paralympic coverage. They acknowledged that media coverage needs to be not only more frequent and sustained, but also more respectful and informed. As illustrated in Figure 2, the most frequently cited suggestions included a focus on athletic performance, avoiding stereotypical narratives, and ensuring equal and consistent coverage. Additional recommendations involved better journalist education—summarised by the expression “do your homework”—and deeper engagement with athletes to produce more holistic and authentic stories.

Below is a summary of the main recommendations that emerged—in the athletes' own words.

#### 4.4.1 “Do your homework”: the call for journalistic preparation

Journalists should strive to understand Paralympic sports—the rules, classification systems, and athletes' backgrounds—so they can report knowledgeably. As one athlete put it, “*we really need technical people to explain to the public what is happening”* (A.15). Having commentators who understand the nuances of the sport (rather than treating the Paralympics as a curiosity) leads to more accurate and insightful storytelling. Reporters were urged to research each athlete's competitive record and context before an interview, ensuring they ask informed questions and avoid basic factual errors.

#### 4.4.2 Focusing on athletic performance, not disability

Nearly every athlete stressed that coverage should center on sporting results, skills, and competition. “*It should just be about the sport,”* one interviewee advised bluntly (A.5). Media should report on times, scores, strategies, and training—just as they would for any sports event—instead of fixating on an athlete's impairment or personal life. Athletes noted that interviews can ask about race strategy or goals for next season instead of repeatedly asking about their disability and forgetting that “*they want to be seen more as athletes and less as people with disabilities”* (A.3). By highlighting athleticism, the media would normalize the image of Paralympians as serious athletes and help shift the focus to their performance.

#### 4.4.3 Avoiding stereotypical narratives

The athletes strongly recommended that journalists steer clear of pitying tones and over-glorifying language. They do not want to see coverage that paints them as tragic figures or as superhuman anomalies. Journalists “*should not put our impairment at the focus on the coverage or news neither to treat it as the core of the story”* one athlete noted (A.16). Disability can be mentioned as context when relevant, but it shouldn't dominate the narrative. The same goes for the incessant “inspirational hero” framing—as discussed, many athletes do not want every report to label them “*inspirational”* simply for living their life. The advice to media is to avoid sensationalizing either difficulties or triumphs; keep the tone balanced and humanizing. In short, coverage should neither patronize nor pedestalize Paralympians, but treat them with the same mix of critique and respect afforded to other athletes.

#### 4.4.4 Ensuring equal and consistent coverage

A recurrent plea was for more continuous media attention beyond the Paralympic Games. Athletes feel that journalists should cover Paralympic sports in the intervening years—major championships, national leagues, record-breaking performances—rather than forgetting about them until the next Paralympics. “*We were super followed [during the Games], which in my opinion is how it should be [all the time]*,”one athlete pointed out (A.9). They want media outlets to commit to regular reporting on Paralympic events and to allocate space in sports pages or broadcasts just as is done for Olympic sports. This also means giving Paralympic achievements equal prominence—for example one of our interviewed athletes mentioned “*Paralympic world record should be celebrated with similar fanfare as an Olympic world record.”*. By integrating Paralympic sport into the regular sports news cycle, the media can build year-round public interest and respect for these athletes.

#### 4.4.5 Inclusive storytelling: engaging directly with athletes

Some athletes suggested that journalists should spend more time with Paralympians to gain deeper insight. Reporters could attend their training sessions, speak with them off-camera, and learn more about the context of their sport. This kind of engagement would help journalists move beyond clichés and uncover more nuanced, “holistic” stories that capture the athlete's personality and experiences. A better understanding of how the sport works (for instance, how classification or competition formats operate) can also help reporters educate audiences, so that viewers appreciate the performances on their own terms. In general, the athletes invite media to see them as partners in representation: if journalists approach with genuine interest in the sport and the athlete's perspective, the Paralympians are eager to collaborate and share their world, resulting in richer and more accurate narratives.

Finally, the Paralympic athletes interviewed conveyed a nuanced understanding of their current media representation. They acknowledged improvements—particularly the growing visibility during the recent Paralympic Games and the empowering possibilities of social media—yet they also identified clear areas for change. Across the themes of coverage, comparisons, and content of stories, a common thread was the desire to be treated “like any other athletes”: given fair coverage, portrayed without bias or cliché, and respected for their sporting prowess. The insights from these athletes underscore the importance of shifting media practices to both increase the quantity of Paralympic sport coverage and enhance its quality.

## 5 Discussion

Although several athletes acknowledged a gradual improvement in visibility—particularly since the London 2012 Games, in line with previous research (1, 2)—this increased attention has not necessarily been accompanied by a qualitative shift in framing. The results of this study indicate that Italian Paralympic athletes generally perceive current media coverage as both insufficient and skewed. Participants reported that media attention remains largely concentrated around major events, and overall exposure remains significantly lower than that afforded to Olympic athletes. Stories too often foreground disability or personal adversity at the expense of athletic achievement. Even as interest in Paralympic sport grows, media portrayals continue to rely on narrow or sensationalist narratives. The representation of disability as inherently extraordinary or tragic remains deeply embedded in public discourse, often preventing Paralympians from being treated as equal protagonists in sport. These findings confirm critiques of disability sport media coverage that has been observed in similar studies conducted in other cultural contexts (53).

A key theme was the persistence of two stereotypical frames—the “supercrip” hero and the pity narrative—which mirror those well-documented in disability media literature. Athletes confirmed frequently encountering these portrayals: being depicted either as inspirational heroes who “overcome” disability or as tragic figures eliciting sympathy (5, 7, 11, 54). Most athletes in this study challenged both frames. They argued that being labeled “heroes” simply for doing sport is patronizing, aligning with scholarly critiques that the supercrip narrative, though intended to inspire, may reinforce condescending attitudes. Likewise, they resented pity-focused stories framing disability as personal tragedy—a medicalized view they reject. Notably, a few individuals expressed nuanced views—for example, one athlete (A.2) appreciated the positive intent behind the hero label even as she recognized its excess—but overall, participants voiced a clear preference for coverage that avoids these extremes and instead portrays them as multi-faceted athletes.

These reactions illustrate Stuart Hall's encoding/decoding model (39, 40). The Paralympians, as consumers of their own media portrayal, often adopted oppositional readings of the dominant media frames—recognizing and actively rejecting the “inspiration” or “pity” framing. Many reframed media stories to highlight athletic accomplishments rather than disability, effectively countering the intended narrative. Some responses were more negotiated: athletes might appreciate a journalist's celebratory tone yet critique the overemphasis on impairment or personal backstory. Virtually none of the athletes fully accepted the media's portrayal at face value. This active decoding underscores Paralympians' agency as audiences: they do not passively receive messages, but filter and contest them through lived experience. Such findings reinforce Hall's model by showing that those with insider knowledge of the subject matter (here, the athletes speaking about themselves) are especially likely to challenge media frames that misalign with their reality.

From a disability representation perspective, the findings reveal a clash between paternalistic framing and the athletes' self-identity. Media focus on impairment and adversity corresponds to a paternalistic or medical model of disability (19, 20), whereas the athletes favor a view closer to the social model—foregrounding their athletic role and treating disability as a natural aspect rather than a defining flaw (21, 23, 24). Their repeated plea to be seen “*like any other athletes”* exemplifies a desire for normalization in coverage. In effect, they are calling for narratives that acknowledge their disability for context but do not let it eclipse their sporting identity. This nuanced stance neither ignores disability nor centers it excessively, aligning with more progressive representation ideals. It confirms prior scholarship urging more complex portrayals and shows that athletes themselves articulate the need for balanced representation.

Regarding how to improve coverage, the athletes offered consistent recommendations that carry clear implications for media practice. They urged journalists to become more educated about Paralympic sports—to “*do their homework”* on rules, classifications, and athletes' backgrounds—so that reporting can be knowledgeable and avoid clichéd depictions. They emphasized focusing on athletic performance rather than disability, urging that Paralympic competitions be covered with the same rigor as other sports (37, 38). Importantly, they warned against stereotypical tropes of pity and super-heroism, advising reporters to tone down sensationalist language and treat Paralympians with the same respect and critical eye given to Olympians. They also pleaded for consistent, year-round coverage to integrate Paralympic sport into mainstream sports discourse. Finally, athletes invited journalists to engage with them more deeply—for instance, by attending training sessions or speaking with them off-camera—to facilitate more authentic and holistic storytelling. Implementing these suggestions could shift media narratives toward greater fairness and accuracy, treating Paralympians as true elite athletes rather than curiosities.

Overall, these insights delineate both the shortcomings in current media representation and concrete ways to improve it. They largely support the exploratory hypotheses: most athletes view present coverage as inadequate and stereotype-laden; those with extensive media exposure often voiced especially critical perspectives; and athletes who engage in self-representation (for example, via social media) demonstrated strong resistance to stereotypes and proactively promoted alternative narratives about their lives (17). This study thus confirms many issues raised in the literature—such as the prevalence of the supercrip and pity frames and the inequality of sports coverage—while contributing the athletes' own evaluations of those issues. Their voices lend credence to calls for change in media practices and highlight that Paralympians are not merely subjects but active shapers of their own representation.

Finally, the findings open avenues for future research. Comparative studies could explore whether Paralympic athletes in other countries share similar perceptions or if different media cultures produce distinct experiences. Longitudinal research might examine how media portrayals and athlete perceptions evolve as awareness of these issues grows—for instance, assessing the impact if media start implementing some of the recommended changes. Additionally, further work could connect these athlete perspectives with audience studies, investigating how the public interprets Paralympic media narratives and whether aligning coverage more closely with athletes' preferred framing improves public understanding. Examining the effects of representation on the athletes themselves (such as on their identity, sponsorship opportunities, or societal recognition) would also be a valuable extension.

Moreover, future research could enhance validity by triangulating athlete perspectives with complementary data sources such as media content analysis or audience reception, offering a more holistic understanding of representation dynamics. Pursuing these directions will further bridge the gap between how disability sport is portrayed and how it is experienced by athletes.

## 6 Conclusion

This research offers a nuanced understanding of how Italian Paralympic athletes view their portrayal in the media. The findings have significant relevance for sports media discourse and disability representation. Athletes report that mainstream coverage of Paralympic sport, while slowly improving, remains limited and inconsistent, with attention peaking during the Games and fading in between. Several athletes pointed out that their accomplishments receive far less media attention than those of Olympic athletes, underscoring a perceived hierarchy in sports coverage. Equally important, they highlighted a qualitative disconnect media narratives tend to oscillate between triumphalist “superhero” tropes and sympathetic disability stories, rather than simply treating Paralympians as athletes. In the athletes' assessment, current coverage still leans toward patronizing depictions rather than truly empowering ones—a dynamic they believe must change.

By centering Paralympic athletes' voices—an approach largely absents from previous research—this study confirms and nuances existing critiques of Paralympic media representation. For instance, it validates the prevalence of the “supercrip” and pity frames while providing firsthand evidence of how these narratives impact athletes' experiences. Athletes describe frustration, identity tension, and the use of strategies like social media to counteract one-dimensional portrayals. These insights enhance theoretical understandings of media and disability, illustrating how athletes actively contest dominant media frames.

The study also broadens the scope of Paralympic media research geographically, suggesting that issues identified in other countries' media are echoed in Italy. Collectively, the contributions lie in bridging sports communication, disability studies, and audience reception. In doing so, this interdisciplinary study shows the benefit of integrating established frameworks with lived experience, ensuring that theoretical debates remain grounded. It exemplifies how including disabled athletes' perspectives can enrich and challenge prevailing paradigms of media representation. Methodologically, this approach shows that in-depth athlete interviews can complement media content analysis, yielding a fuller picture of how representation is produced and received.

The findings offer actionable recommendations for sports media professionals, organizations, and policymakers to foster a more inclusive and balanced media landscape. This includes adopting reporting practices that move beyond stereotypical portrayals and promote a view of disabled athletes as equal participants in sport. More broadly, the study's insights reinforce the importance of authentic representation for any marginalized group.

## Data Availability

The raw data supporting the conclusions of this article will be made available by the authors, without undue reservation.
